# Baseline Serum Potassium Within the Normal Range Is Independently Associated With One-Year Myocardial Strain Recovery in Patients With ST-Segment Elevation Myocardial Infarction Treated With Primary Percutaneous Coronary Intervention: A Cardiac Magnetic Resonance Study

**DOI:** 10.31083/RCM48335

**Published:** 2026-08-13

**Authors:** Shancheng Wang, Jinhua Zhang, Yu Zhang, Ding Wang, Xianping Meng, Junwei Xu, Wei Xing

**Affiliations:** ^1^Department of Radiology, The Affiliated Jiangyin Hospital of Nantong University, Jiangyin Clinical College of Xuzhou Medical University, Jiangyin People’s Hospital, 214400 Jiangyin, Jiangsu, China; ^2^Department of Cardiology, Nanjing Chest Hospital, Affiliated Nanjing Brain Hospital, Nanjing Medical University, 210029 Nanjing, Jiangsu, China; ^3^Department of Radiology, The Third Affiliated Hospital of Soochow University, 213001 Changzhou, Jiangsu, China

**Keywords:** potassium, ST elevation myocardial infarction, magnetic resonance imaging, global longitudinal strain, ventricular remodeling

## Abstract

**Background:**

While serum potassium abnormalities predict mortality in acute myocardial infarction, whether potassium levels within the normal range influence long-term myocardial functional recovery remains unknown. Therefore, this study aimed to investigate the association between baseline potassium levels and 1-year cardiac magnetic resonance (CMR)-derived global longitudinal strain in patients with ST-segment elevation myocardial infarction (STEMI).

**Methods:**

This retrospective cohort study enrolled 120 consecutive patients with STEMI (mean age 52.4 ± 12.6 years, 94.2% male) who underwent primary percutaneous coronary intervention (PCI) and completed 1-year CMR follow-up. Baseline electrolyte levels, including potassium, sodium, chloride, magnesium, ionized calcium, and phosphorus, were measured before the intervention. The primary outcome was 1-year absolute global longitudinal strain (abs_GLS) assessed by CMR feature tracking. Analysis of covariance (ANCOVA) was used to evaluate associations between electrolyte levels and myocardial strain after adjustment for baseline abs_GLS and 16 clinical covariates, including discharge medications.

**Results:**

Baseline potassium averaged 3.92 ± 0.26 mmol/L, with 90% of values ranging from 3.42 to 4.39 mmol/L. After adjustment for baseline abs_GLS and 16 clinical covariates, potassium was the only electrolyte significantly associated with 1-year abs_GLS in the prespecified primary model. Each 1-SD increase in potassium (0.26 mmol/L) was associated with a 0.782 percentage-point higher 1-year abs_GLS (95% CI: 0.042–1.521, *p *= 0.041). Categorical analysis showed a nonsignificant trend across potassium tertiles (T1 [<3.82 mmol/L]: 10.3%; T2 [3.82–4.02 mmol/L]: 11.6%; T3 [>4.02 mmol/L]: 12.0%; *p* for trend = 0.159), with the highest tertile exhibiting a 1.7-percentage-point higher adjusted abs_GLS than the lowest tertile.

**Conclusions:**

Among patients with STEMI treated with primary PCI, baseline serum potassium, even within the normal range, is independently associated with 1-year myocardial strain recovery, with a trend toward superior outcomes at higher-normal levels. These findings suggest that potassium may serve as a readily available prognostic biomarker for risk stratification; nonetheless, randomized trials are warranted to investigate whether targeted optimization of potassium can improve post-infarction cardiac recovery.

## 1. Introduction

ST-segment elevation myocardial infarction (STEMI) remains a leading cause of morbidity and mortality worldwide [1]. While timely primary percutaneous coronary intervention (PCI) has improved acute survival, up to 25% of patients develop adverse left ventricular (LV) remodeling—characterized by ventricular dilatation, wall thinning, and progressive contractile dysfunction—leading to post-infarction heart failure with substantially worse long-term outcomes [2,3,4]. Accurate prognostic stratification to identify patients at high risk for incomplete myocardial recovery is critical for guiding intensive surveillance and optimizing therapy [5].

Electrolyte homeostasis, particularly serum potassium, has emerged as an important prognostic factor in acute myocardial infarction. Landmark studies have demonstrated a U-shaped association between potassium levels and mortality in acute myocardial infarction (AMI) patients, with both hypokalemia (<3.5 mmol/L) and hyperkalemia (≥4.5 mmol/L) independently predicting increased risk of death and major adverse cardiovascular events (MACE) [6,7,8]. These associations are mechanistically plausible given potassium’s fundamental role in cardiac electrophysiological stability through regulation of ion channel conductance and action potential repolarization [9]. However, existing evidence has focused almost exclusively on hard clinical endpoints (mortality, arrhythmia), with limited investigation into whether potassium levels influence myocardial functional recovery following successful revascularization.

Cardiac magnetic resonance (CMR) imaging with myocardial strain analysis has emerged as a powerful tool for characterizing post-infarction ventricular function. Global longitudinal strain (GLS) quantifies the percentage shortening of the left ventricle along its longitudinal axis during systole and provides superior sensitivity for detecting subclinical myocardial dysfunction compared to traditional left ventricular ejection fraction (LVEF) [10,11]. Recent studies have demonstrated that CMR-derived GLS independently predicts MACE, heart failure hospitalization, and all-cause mortality in STEMI patients [10,12,13]. Despite this prognostic value and the established link between potassium and AMI outcomes, no prior study has examined whether baseline potassium levels—particularly within the normal physiological range—are associated with myocardial strain recovery in STEMI patients. This knowledge gap is clinically significant because, unlike genetic or fixed anatomic factors, potassium homeostasis is potentially modifiable through dietary counseling, oral supplementation, or pharmacological agents.

In this hypothesis-driven observational study guided by prior evidence demonstrating potassium’s U-shaped association with mortality in AMI [6], we sought to investigate the association between baseline serum potassium concentration and 1-year myocardial strain recovery in patients with STEMI treated with primary PCI. Given the established prognostic relevance of potassium in AMI mortality studies, we hypothesized that higher potassium levels within the normal range would be independently associated with superior functional recovery. We additionally explored associations of five other routinely measured electrolytes (sodium, chloride, magnesium, ionized calcium, phosphorus) with strain outcomes. The findings may inform risk stratification algorithms and generate hypotheses for future interventional trials evaluating targeted potassium optimization to mitigate adverse ventricular remodeling in STEMI patients.

## 2. Materials and Methods

### 2.1 Study Design and Population

This retrospective cohort study enrolled consecutive STEMI patients who underwent primary PCI at the Affiliated Jiangyin Hospital of Nantong University between January 2020 and December 2023. The study was approved by the institutional ethics committee (Approval No. 2025-Lun-Shen-Yan-014) and conducted in accordance with the Declaration of Helsinki. Written informed consent was waived due to the retrospective design and use of anonymized data.

Eligible participants were adults (≥18 years) with STEMI diagnosed per the Fourth Universal Definition of Myocardial Infarction (ST-elevation ≥1 mm in ≥2 contiguous leads or new left bundle branch block, plus elevated troponin). All patients underwent successful primary PCI (Thrombolysis in Myocardial Infarction (TIMI) flow grade 3 post-procedure) and had complete baseline electrolyte measurements (obtained pre-PCI), baseline CMR within 7 days post-PCI, and 1-year follow-up CMR (12 months ± 2 weeks). Exclusion criteria included prior MI or revascularization, severe renal dysfunction (estimated glomerular filtration rate (eGFR) <30 mL/min/1.73 m^2^), active malignancy, chronic inflammation, CMR contraindications, inadequate image quality, or death/loss to follow-up before 12 months.

A total of 120 patients met all eligibility criteria and comprised the final analytic cohort (**Supplementary Fig. 1**).

### 2.2 Clinical and Laboratory Data

Baseline demographics, cardiovascular risk factors (diabetes, hypertension, smoking), Killip class, vital signs, and symptom-to-balloon time were extracted from electronic medical records. Symptom-to-balloon time was defined as the interval from patient-reported symptom onset to first balloon inflation and was expressed in hours. This variable represents total ischemic time, including prehospital delay, and should not be interpreted as in-hospital door-to-balloon time. Discharge medications (renin-angiotensin system inhibitors (RASi), β-blockers, and mineralocorticoid receptor antagonists (MRA)) were recorded as part of guideline-directed medical therapy assessment. Fasting venous blood samples were collected at admission (pre-PCI). Electrolytes (sodium, potassium, chloride, magnesium, ionized calcium, phosphorus) were measured using the ion-selective electrode method. Serum albumin was assayed by the bromocresol green method, and eGFR was calculated via the Chronic Kidney Disease Epidemiology Collaboration (CKD-EPI) equation.

### 2.3 Exposure Variables

#### 2.3.1 Primary Exposure: Potassium

Based on prior evidence linking potassium to AMI outcomes, baseline serum potassium was the primary exposure. It was analyzed both as a continuous variable (per mmol/L) and categorically using tertiles:

T1 (lower-normal): <3.82 mmol/L (n = 40)

T2 (mid-normal): 3.82–4.02 mmol/L (n = 40)

T3 (upper-normal): >4.02 mmol/L (n = 40)

Tertile grouping was used only for secondary exploratory presentation, whereas the continuous potassium analysis remained primary.

#### 2.3.2 Secondary Exposures

Five additional electrolytes (sodium, chloride, magnesium, ionized calcium, phosphorus) were evaluated as exploratory exposures, analyzed only as continuous variables.

### 2.4 CMR Imaging and Strain Analysis

CMR was performed using a Philips Ingenia 3.0-T scanner equipped with a dStream Torso coil (Philips Healthcare, Best, the Netherlands). ECG-gated, breath-hold balanced turbo field echo (TFE) sequences were used. Standard long-axis (2-, 3-, and 4-chamber) views and short-axis cine stacks were acquired. Baseline CMR was performed within 7 days after PCI (median, 5 days; interquartile range (IQR), 4–6 days), and follow-up CMR was performed at 12 months (±2 weeks) using an identical imaging protocol.

 Strain analysis: GLS was derived offline from long-axis cine images using feature-tracking software (QStrain version 3.2, Medis Medical Imaging Systems B.V., Leiden, the Netherlands). Endocardial borders were manually traced at end-diastole; myocardial tracking was automated and visually verified. By convention, systolic GLS is negative because it represents longitudinal myocardial shortening. For all study-specific analyses, tables, and figures, we used the absolute magnitude of signed GLS (abs_GLS = |GLS|), expressed as a positive percentage. Thus, a signed GLS value of −15% is reported as an abs_GLS value of 15%, and a higher abs_GLS indicates greater systolic shortening and better myocardial function. Published signed GLS reference values of approximately –20% to –15% correspond to abs_GLS values of approximately 15% to 20% [14]. Inter- and intra-observer intraclass correlation coefficients (ICC) were 0.88 and 0.94, respectively.

Published reference values for CMR feature-tracking-derived GLS are conventionally reported using signed values, with normal systolic GLS generally ranging from approximately −20% to −15%. Under the absolute-value convention used in this study, this signed range corresponds to abs_GLS values of approximately 15% to 20%, whereas less negative signed GLS values (e.g., >−15%) correspond to lower abs_GLS values (e.g., <15%) and indicate impaired systolic function.

### 2.5 Outcome Definition

The primary outcome was 1-year absolute GLS (follow-up abs_GLS) from the 12-month CMR. In analysis of covariance (ANCOVA) models, baseline abs_GLS was included as a covariate to adjust for initial myocardial function. Δabs_GLS was defined as 1-year abs_GLS minus baseline abs_GLS and was expressed in percentage points.

### 2.6 Statistical Analysis

#### 2.6.1 Sample Size and Data Quality

The cohort of 120 patients with 17 model covariates (baseline abs_GLS + 16 clinical variables) yielded a 7.1:1 events-per-variable ratio, meeting recommended thresholds for stable regression estimates. All variables had complete data (0% missing).

#### 2.6.2 Descriptive Statistics

Continuous variables were assessed for normality and presented as mean ± standard deviation (SD) or median (IQR). Categorical variables are reported as n (%). Baseline characteristics were compared across potassium tertiles using analysis of variance (ANOVA) (or Kruskal-Wallis) for continuous variables and chi-square (or Fisher’s exact) test for categorical variables.

#### 2.6.3 Univariable Analysis

Pearson correlation was used to assess univariable associations between each baseline electrolyte concentration and Δabs_GLS. For potassium tertiles, one-way ANOVA compared unadjusted outcomes, with post-hoc pairwise comparisons (Tukey HSD) and linear trend tests.

#### 2.6.4 Multivariable ANCOVA

ANCOVA evaluated independent associations between electrolytes and 1-year abs_GLS, adjusting for baseline abs_GLS and 16 clinical covariates (age, gender, diabetes, hypertension, smoking, Killip class, eGFR, systolic blood pressure (SBP), diastolic blood pressure (DBP), heart rate, symptom-to-balloon time, albumin, culprit artery, and discharge medications (RASi, β-blockers, and MRA)).

Model specifications:

Model 1 (primary analysis): Each electrolyte was entered separately as a standardized continuous exposure, with adjustment for baseline abs_GLS and 16 clinical covariates. For potassium, a complementary tertile-based analysis was also performed. Model 2 (robustness analysis): All six standardized electrolyte exposures were entered simultaneously with the same covariates to evaluate whether the potassium association remained robust after mutual adjustment for the other electrolytes.

For categorical potassium analysis, adjusted marginal means were computed using the emmeans package, with T1 as reference. Linear trend tests assessed dose-response across ordered tertiles.

Model diagnostics (multicollinearity, homoscedasticity, normality) were verified. Continuous electrolyte variables were standardized to 1 SD before model fitting, whereas the outcome, 1-year abs_GLS, remained on its original percentage-point scale. Accordingly, β coefficients represent the percentage-point change in 1-year abs_GLS per 1-SD increase in electrolyte concentration. Model fit is summarized by adjusted R^2^.

Methodological justification for tertile categorization:

Serum potassium levels were analyzed both as a continuous variable and categorized into tertiles to facilitate clinical interpretation and evaluate potential dose-response relationships. Continuous potassium levels were standardized (per 1-standard deviation increase) for regression analyses to allow comparison of effect sizes across different electrolytes.

Potassium was analyzed primarily as a continuous variable to preserve statistical information. Tertile categorization was used only as a secondary exploratory analysis to facilitate clinical interpretation and describe differences across the observed potassium distribution. Tertile cutoffs were defined from the sample distribution, yielding 40 patients per group [15]. Because categorizing a continuous exposure can result in loss of information and statistical power [16], the categorical findings were interpreted cautiously and were not considered evidence of validated clinical thresholds.

To address potential concerns about information loss from categorization, we performed sensitivity analyses treating potassium as a continuous variable in all multivariable models. Restricted cubic splines with 3 knots were used to test for non-linearity in the potassium-outcome relationship. The consistency of findings across continuous and categorical analyses strengthens the robustness of our conclusions.

#### 2.6.5 Nonlinearity Testing

Restricted cubic splines (RCS) with three knots were used to test for nonlinear electrolyte-outcome relationships. Wald tests evaluated nonlinearity (*p* < 0.05 indicates significant deviation from linearity).

#### 2.6.6 Stratified Analysis

Given left anterior descending (LAD) artery predominance (77.5%), stratified ANCOVA models were fitted for LAD vs. non-LAD subgroups, adjusting for baseline abs_GLS and the same 16 clinical covariates (including discharge medications). Interaction terms (Electrolyte × LAD) were tested; false discovery rate (FDR) correction was applied (*p* < 0.05 for significance).

#### 2.6.7 Sensitivity Analyses

Robustness was assessed by: (1) excluding extreme potassium (<3.0 or >5.0 mmol/L); (2) dichotomizing potassium at the median (3.94 mmol/L) based on the study population distribution; (3) dichotomizing at 4.0 mmol/L (literature-based threshold from prior acute myocardial infarction studies). All sensitivity analyses used the same multivariable ANCOVA framework adjusting for baseline abs_GLS and 16 clinical covariates (including discharge medications). Results are in **Supplementary Table 1**.

#### 2.6.8 Software

All statistical analyses were performed using R version 4.3.0 (R Foundation for Statistical Computing, Vienna, Austria) within the RStudio integrated development environment (Posit Software, PBC, Boston, MA, USA). All tests were two-sided, and a *p* value < 0.05 was considered statistically significant; FDR correction was applied to interaction analyses.

## 3. Results

### 3.1 Study Population and Baseline Characteristics

A total of 120 STEMI patients (mean age 52.4 ± 12.6 years, 94.2% male) who completed primary PCI and 1-year CMR follow-up were analyzed. The cohort exhibited typical risk profiles: 63.3% had hypertension, 62.5% were current smokers, and 41.7% had diabetes mellitus. Most patients presented with Killip class I (70.0%), and the LAD was the culprit vessel in 77.5% of cases. Guideline-directed medical therapy at discharge included renin-angiotensin system inhibitors (68.3%), β-blockers (75.0%), and mineralocorticoid receptor antagonists (40.8%).

Baseline electrolyte levels were predominantly within normal ranges, with potassium averaging 3.92 ± 0.26 mmol/L (90% of patients between 3.42–4.39 mmol/L) (**Supplementary Fig. 2**). The mean baseline abs_GLS was 11.1 ± 4.6%, improving modestly to 11.6 ± 6.0% at 1-year follow-up (Δabs_GLS: 0.50 ± 3.92 percentage points), indicating substantial inter-individual variability in functional recovery.

To facilitate categorical analysis, patients were stratified by potassium tertiles: T1 (<3.82 mmol/L, n = 40), T2 (3.82–4.02 mmol/L, n = 40), and T3 (>4.02 mmol/L, n = 40). Table 1 demonstrates that, apart from serum potassium—the variable used to define the tertile groups—all demographic, clinical, and laboratory variables and discharge medications were well balanced across groups (all *p* > 0.05). As expected from the tertile definition, serum potassium concentrations differed substantially across groups (*p *< 0.001). Baseline abs_GLS showed no significant difference across tertiles (T1: 11.89%, T2: 10.93%, T3: 10.51%; *p* = 0.390). Similarly, unadjusted 1-year abs_GLS showed no significant difference (T1: 11.10%, T2: 12.04%, T3: 11.71%; *p* = 0.781).

**Table 1.** **Baseline characteristics and 1-year myocardial strain outcomes according to baseline serum potassium tertiles**.

Variable		T1 (n = 40)	T2 (n = 40)	T3 (n = 40)	*p* value
Demographics					
	Age, years	50.2 ± 11.8	52.2 ± 15.3	54.8 ± 10.0	0.273
	Male, n (%)	39 (97.5%)	38 (95.0%)	36 (90.0%)	0.346
Cardiovascular risk factors					
	Diabetes mellitus, n (%)	13 (32.5%)	21 (52.5%)	16 (40.0%)	0.186
	Hypertension, n (%)	23 (57.5%)	27 (67.5%)	26 (65.0%)	0.627
	Current smoking, n (%)	25 (62.5%)	25 (62.5%)	25 (62.5%)	1
Clinical characteristics					
	Killip class I/II/III, n	25/11/4	31/8/1	28/10/2	0.537
	Systolic BP, mmHg	139.0 ± 24.1	133.9 ± 17.0	131.6 ± 20.9	0.271
	Diastolic BP, mmHg	86.9 ± 14.4	86.9 ± 15.0	81.6 ± 13.6	0.166
	Heart rate, bpm	72.2 ± 12.9	70.5 ± 12.4	70.8 ± 11.1	0.802
	eGFR, mL/min/1.73 m^2^	105.8 ± 23.4	104.5 ± 19.1	107.2 ± 23.4	0.864
	Symptom-to-balloon time, hours	15.5 (3.0, 68.2)	9.0 (3.0, 24.8)	24.0 (4.0, 121.2)	0.157
Laboratory parameters					
	Potassium, mmol/L	3.63 ± 0.16	3.92 ± 0.08	4.21 ± 0.11	<0.001
	Sodium, mmol/L	139.3 ± 1.9	139.7 ± 1.7	139.6 ± 2.2	0.593
	Chloride, mmol/L	103.3 ± 3.8	102.7 ± 3.1	103.2 ± 3.9	0.694
	Magnesium, mmol/L	0.90 ± 0.03	0.90 ± 0.04	0.91 ± 0.04	0.718
	Ionized calcium, mmol/L	0.96 ± 0.20	1.01 ± 0.20	1.07 ± 0.21	0.051
	Phosphorus, mmol/L	0.94 ± 0.12	0.93 ± 0.11	0.97 ± 0.10	0.239
	Albumin, g/L	42.1 ± 3.9	40.9 ± 3.6	40.7 ± 3.8	0.211
CMR parameters					
	LAD as culprit vessel, n (%)	29 (72.5%)	34 (85.0%)	30 (75.0%)	0.367
	Baseline abs_GLS, %	11.89 ± 4.82	10.93 ± 4.24	10.51 ± 4.70	0.390
	1-year abs_GLS, %	11.10 ± 4.98	12.04 ± 6.76	11.71 ± 6.20	0.781
	Δabs_GLS, %	–0.9 (–2.5, 0.5)	0.3 (–2.0, 2.3)	0.5 (–2.1, 2.8)	0.063
Medications at discharge					
	RASi, n (%)	25 (62.5%)	31 (77.5%)	26 (65.0%)	0.303
	β-blocker, n (%)	27 (67.5%)	33 (82.5%)	30 (75.0%)	0.301
	MRA, n (%)	20 (50.0%)	14 (35.0%)	15 (37.5%)	0.343

This table presents baseline demographic, clinical, laboratory, angiographic, and cardiac magnetic resonance (CMR) characteristics, together with 1-year myocardial strain outcomes, for 120 patients with ST-segment elevation myocardial infarction (STEMI) stratified by baseline serum potassium tertiles: T1 (lower-normal, <3.82 mmol/L, n = 40), T2 (mid-normal, 3.82–4.02 mmol/L, n = 40), and T3 (upper-normal, >4.02 mmol/L, n = 40). Demographic variables include age and gender. Cardiovascular risk factors include diabetes mellitus, hypertension, and current smoking status. Clinical characteristics include Killip class, systolic and diastolic blood pressure (BP), heart rate, estimated glomerular filtration rate (eGFR), and symptom-to-balloon time. Laboratory parameters include all six baseline electrolytes (potassium, sodium, chloride, magnesium, ionized calcium, phosphorus) and albumin. CMR parameters include culprit vessel (left anterior descending artery (LAD)), baseline absolute global longitudinal strain (abs_GLS) measured within 7 days post-percutaneous coronary intervention (PCI), 1-year follow-up abs_GLS, and change in abs_GLS (Δabs_GLS = follow-up minus baseline). Normally distributed continuous variables are presented as mean ± standard deviation (SD), whereas symptom-to-balloon time and Δabs_GLS are presented as median (interquartile range (IQR)). Categorical variables are presented as n (%). *p *values were obtained using one-way analysis of variance (ANOVA) for normally distributed continuous variables, the Kruskal-Wallis test for symptom-to-balloon time and Δabs_GLS, and the chi-square test for categorical variables. The table demonstrates excellent baseline balance across all demographic, clinical, and laboratory variables (all *p* > 0.05), validating the comparability of groups. As expected from the tertile definition, serum potassium concentrations differed substantially across groups (*p* < 0.001). Notably, baseline abs_GLS showed no significant difference (*p* = 0.390), and Δabs_GLS showed no significant gradient in non-parametric analysis (*p* = 0.063), though higher tertiles showed directionally greater functional improvement. Additionally, the three medication variables (RASi, β-blocker, MRA) showed no significant differences across potassium tertiles (all *p* > 0.05), indicating that these potential confounders were evenly distributed and can be appropriately adjusted for in multivariate analysis to confirm the independent prognostic value of potassium. Abbreviations: MRA, mineralocorticoid receptor antagonists; RASi, renin-angiotensin system inhibitors.

Δabs_GLS demonstrated a nonsignificant numerical trend across tertiles (T1: −0.9 [−2.5, 0.5]; T2: 0.3 [−2.0, 2.3]; T3: 0.5 [−2.1, 2.8] percentage points; Kruskal–Wallis *p *= 0.063). After adjusting for baseline abs_GLS and 16 clinical covariates (including age, gender, cardiovascular risk factors, hemodynamic parameters, renal function, culprit vessel, time to reperfusion, albumin, and discharge medications) using ANCOVA, the adjusted 1-year abs_GLS (estimated marginal means) showed a consistent numerical trend across tertiles (T1: 10.3%, T2: 11.6%, T3: 12.0%; *p* for trend = 0.159), with the adjusted mean in the highest tertile being 1.7 percentage points higher than that in the lowest tertile, although the trend did not reach statistical significance. Thus, the unadjusted and adjusted analyses showed directionally consistent but nonsignificant numerical trends across tertiles.

### 3.2 Potassium is Independently Associated With 1-Year Myocardial Strain

#### 3.2.1 Univariable Analysis

In univariable analysis, baseline potassium was positively correlated with Δabs_GLS (r = 0.218, *p* = 0.017; **Supplementary Fig. 3** and **Supplementary Table 2**), whereas linear trend testing across potassium tertiles showed no significant trend (*p* for trend = 0.159).

#### 3.2.2 Multivariable ANCOVA Models

In the prespecified primary analysis (Model 1), each electrolyte was evaluated separately after adjustment for baseline abs_GLS and 16 clinical covariates. Potassium was the only electrolyte significantly associated with 1-year abs_GLS (Table 2). Each 1-SD increase in potassium (0.26 mmol/L) was associated with a 0.782-percentage-point increase in 1-year abs_GLS (95% CI: 0.042–1.521, *p* = 0.041), equivalent to approximately 3.01 percentage points per 1 mmol/L increase. All other electrolytes showed no significant associations (all *p *> 0.40).

**Table 2.** **Multivariable analysis of covariance results for associations between baseline electrolytes and 1-year myocardial strain**.

Electrolyte	Model 1 (primary): β (95% CI)	*p* value	Model 2 (mutually adjusted robustness analysis): β (95% CI)	*p* value
Na	–0.338 (–1.125, 0.450)	0.403	–0.321 (–1.111, 0.470)	0.428
K	0.782 (0.042, 1.521)	0.041	0.866 (0.072, 1.660)	0.035*
Cl	0.239 (–0.587, 1.065)	0.572	0.202 (–0.629, 1.034)	0.635
Mg	–0.172 (–0.960, 0.615)	0.670	–0.299 (–1.109, 0.511)	0.472
Ca_ionized	0.042 (–0.721, 0.805)	0.914	–0.180 (–0.999, 0.638)	0.667
P	0.116 (–0.638, 0.869)	0.764	0.057 (–0.730, 0.845)	0.887

Multivariable analysis of covariance results for associations between baseline electrolytes and 1-year myocardial strain. This table presents β coefficients from multivariable analysis of covariance (ANCOVA) models examining the independent associations between six baseline serum electrolytes and 1-year absolute global longitudinal strain (abs_GLS) recovery in 120 STEMI patients. Model 1 analyzes each electrolyte separately, while Model 2 analyzes all electrolytes simultaneously to account for intercorrelations. All models were adjusted for baseline abs_GLS and the following clinical covariates: age, gender, diabetes mellitus, hypertension, smoking status, Killip class, estimated glomerular filtration rate (eGFR), systolic and diastolic blood pressure, heart rate, serum albumin, culprit vessel (LAD), symptom-to-balloon time, and discharge medications including renin-angiotensin system inhibitors (RASi), β-blockers, and mineralocorticoid receptor antagonists (MRA). The inclusion of discharge medications addresses a key methodological concern regarding potential confounding by guideline-directed medical therapy, as these agents can both improve myocardial strain and affect serum potassium levels. Values are β coefficients with 95% confidence intervals and represent the change in 1-year abs_GLS, expressed in percentage points, per 1-SD increase in baseline electrolyte concentration. Electrolyte exposures were standardized, whereas the outcome was retained on its original scale. *p < 0.05 indicates statistical significance.

Categorical analysis demonstrated a consistent pattern: adjusted marginal means (estimated from ANCOVA models) for 1-year abs_GLS showed an increasing trend across tertiles (T1: 10.3%, T2: 11.6%, T3: 12.0%), with the highest tertile exhibiting a 1.7 percentage-point difference compared to the lowest (linear trend *p* = 0.159). While this trend did not reach conventional statistical significance (*p* < 0.05), the direction and magnitude were consistent with the significant continuous variable analysis (*p* = 0.041), providing complementary evidence for the potassium-strain relationship.

In the robustness analysis (Model 2), in which all six electrolytes were entered simultaneously, the association between potassium and 1-year abs_GLS remained significant (β = 0.866, 95% CI: 0.072–1.660, *p* = 0.035), supporting the robustness of the primary Model 1 finding after mutual adjustment for the other electrolytes. Model diagnostics were satisfactory (adjusted R^2^ = 0.196).

**Fig. 1.**
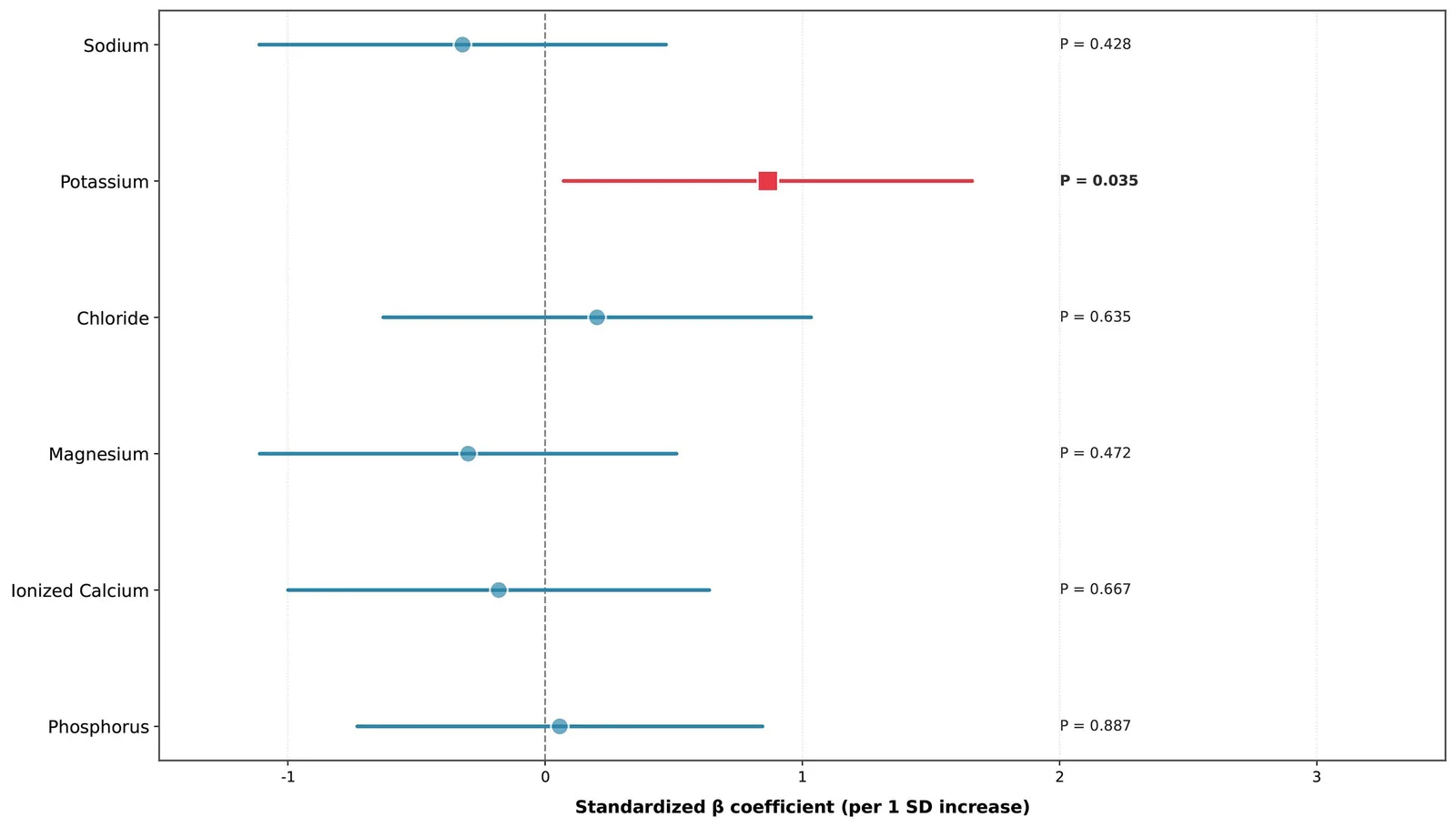
**Multivariable-adjusted associations between baseline electrolytes and 1-year myocardial strain in STEMI patients**. This forest plot displays the independent associations between six baseline serum electrolytes (potassium, sodium, chloride, magnesium, ionized calcium, phosphorus) and absolute global longitudinal strain (abs_GLS) at 1-year follow-up in 120 ST-segment elevation myocardial infarction (STEMI) patients treated with primary percutaneous coronary intervention (PCI). The horizontal axis represents standardized β coefficients (per 1 standard deviation increase in electrolyte concentration), and the vertical axis lists each electrolyte type. Blue circles represent nonsignificant point estimates from multivariable analysis of covariance (ANCOVA) models adjusted for baseline abs_GLS and 16 clinical covariates (age, gender, diabetes, hypertension, smoking, Killip class, estimated glomerular filtration rate, systolic blood pressure, diastolic blood pressure, heart rate, symptom-to-balloon time, albumin, culprit artery, and discharge medications including renin-angiotensin system inhibitors, β-blockers, and mineralocorticoid receptor antagonists), whereas the red square represents the statistically significant potassium estimate. Horizontal lines represent 95% confidence intervals (CI). The dashed vertical line marks the null reference line (β = 0). Potassium was the only electrolyte demonstrating a significant positive association (β = 0.866, 95% CI: 0.072–1.660, *p* = 0.035), indicating that each 1-SD increase (0.26 mmol/L) in baseline potassium was associated with a 0.866 percentage-point improvement in 1-year abs_GLS. All other electrolytes showed no significant associations (all *p* > 0.40).

The multivariable-adjusted associations between all six baseline electrolytes and 1-year myocardial strain are visualized in Fig. 1, which clearly demonstrates that potassium was the only electrolyte with a significant independent association (β = 0.866, 95% CI: 0.072–1.660, *p* = 0.035).

### 3.3 The Relationship Appears Approximately Linear and Consistent Across Subgroups

**Fig. 2.**
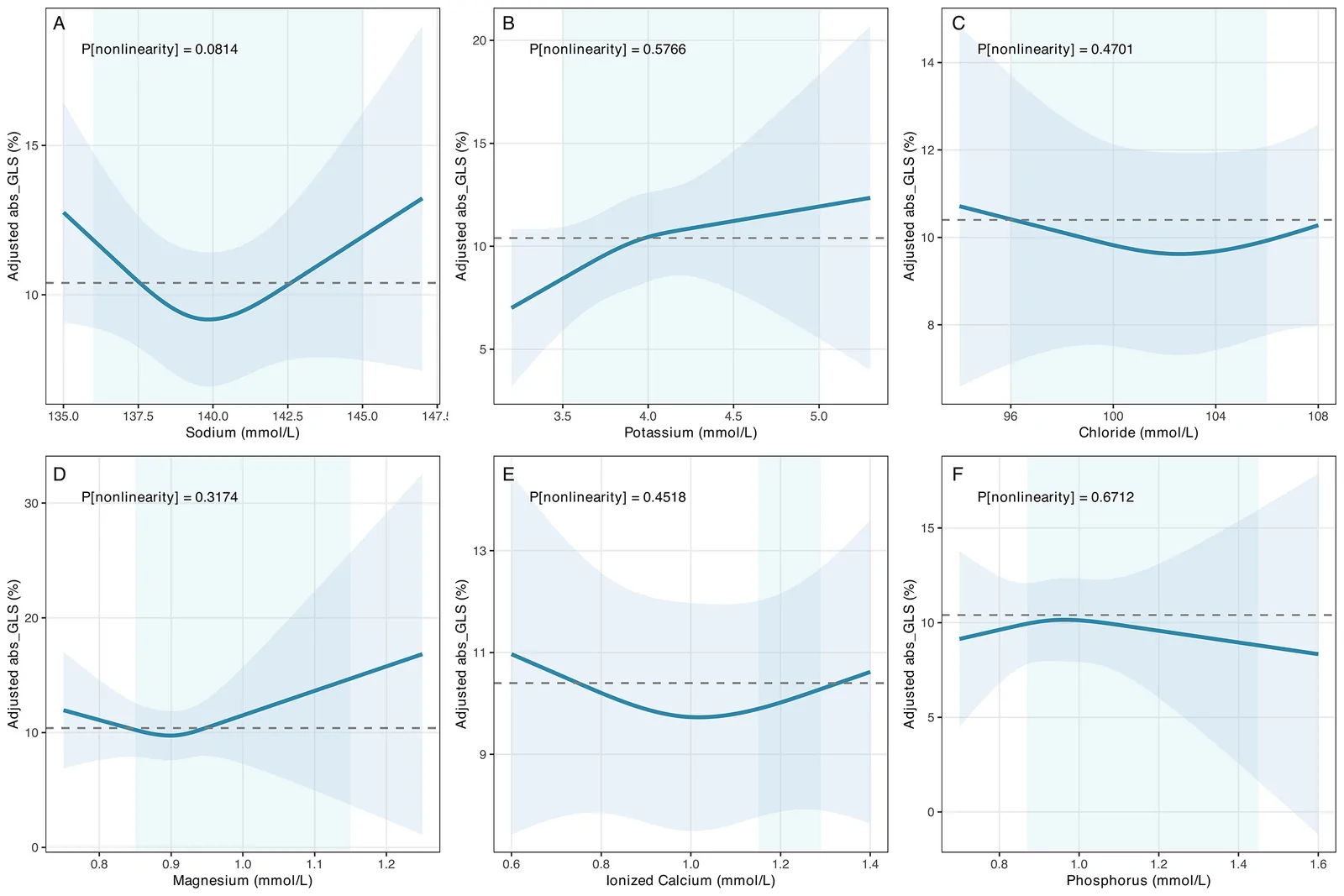
**Dose-response relationships between baseline electrolytes and 1-year myocardial strain in STEMI patients**. This figure presents restricted cubic spline (RCS) analysis with three knots to evaluate potential nonlinear dose-response relationships between six baseline electrolyte concentrations and adjusted 1-year absolute global longitudinal strain (abs_GLS) in 120 STEMI patients. The figure consists of six panels: (A) sodium (mmol/L), (B) potassium (mmol/L), (C) chloride (mmol/L), (D) magnesium (mmol/L), (E) ionized calcium (mmol/L), and (F) phosphorus (mmol/L). For each panel, the horizontal axis displays the respective electrolyte concentration, and the vertical axis represents adjusted 1-year abs_GLS (%). The blue solid line represents the predicted abs_GLS from the RCS model, adjusted for baseline abs_GLS and 16 clinical covariates. The light blue shaded area represents the 95% confidence interval. Wald tests detected no significant nonlinearity for any electrolyte at the conventional 0.05 significance level (all *p*_nonlinearity > 0.05), including potassium (*p*_nonlinearity = 0.5766), suggesting approximately linear relationships within the observed concentration ranges (predominantly normal physiological ranges). The potassium panel (B) demonstrates a consistent positive association across the range of 3.4–4.5 mmol/L, contrasting with the U-shaped mortality associations reported in prior acute myocardial infarction studies.

Restricted cubic spline analysis (Fig. 2) showed no statistically significant evidence of nonlinear associations between electrolyte levels and abs_GLS at the 0.05 significance level. For potassium, the relationship with 1-year abs_GLS showed no evidence of significant nonlinearity (*p*_nonlinearity = 0.5766), contrasting with U-shaped associations previously reported for potassium and mortality. This suggests that, within the predominantly normal range observed here (3.4–4.5 mmol/L), the association between higher potassium and functional recovery is approximately linear. Notably, the confidence intervals widen at higher potassium levels (>4.2 mmol/L), reflecting increased estimation uncertainty due to sparse sample size in this tail region.

Stratified analyses by culprit vessel revealed consistent potassium effects in both LAD (β = 2.48, *p* = 0.164) and non-LAD subgroups (β = 5.19, *p* = 0.058), though statistical power was limited in the smaller non-LAD cohort (n = 27). Formal interaction testing showed no significant effect modification (*p*_interaction = 0.475), indicating that the potassium-strain association does not differ by infarct location.

## 4. Discussion

### 4.1 Principal Findings

This study demonstrates that baseline serum potassium, even within the normal physiological range, is independently associated with 1-year myocardial strain recovery in STEMI patients treated with primary PCI. Among the six electrolytes examined, potassium was the only electrolyte significantly associated with 1-year abs_GLS in the prespecified primary analysis (Model 1). Each 1-SD increase in potassium (0.26 mmol/L) was associated with a 0.782-percentage-point increase in 1-year abs_GLS (95% CI: 0.042–1.521, *p* = 0.041) after adjustment for baseline abs_GLS and 16 clinical covariates. The association was also observed in univariable analysis (r = 0.218, *p* = 0.017) and remained significant in the mutually adjusted Model 2 robustness analysis (β = 0.866, 95% CI: 0.072–1.660, *p* = 0.035). The relationship appeared approximately linear across tertiles spanning 3.5 to 4.3 mmol/L, though the categorical trend analysis showed no significant association (*p* for trend = 0.159). This appears to be the first study linking baseline potassium concentration with CMR-derived functional recovery in acute myocardial infarction, extending its established prognostic value beyond hard endpoints (mortality, arrhythmia) to a quantitative measure of ventricular remodeling. These findings raise the hypothesis that optimizing potassium levels may represent a modifiable target for improving post-infarction cardiac function, though randomized trials are needed to establish causality.

### 4.2 Comparison With Previous Studies

The prognostic significance of potassium in acute myocardial infarction has been extensively documented in large-scale observational studies, predominantly focusing on mortality and arrhythmia. Goyal et al. [6] analyzed 38,689 AMI patients and identified a U-shaped relationship between potassium and in-hospital mortality, with optimal survival at 3.5 to <4.5 mmol/L. Krogager et al. [17] demonstrated in 2596 post-MI patients that potassium levels outside 3.9–4.5 mmol/L were associated with elevated mortality risk (HR 1.84 for 3.5–3.8 mmol/L). Recent 2024 evidence confirmed that hypokalemia nearly doubled mortality risk (RR 1.69) and hyperkalemia tripled it (RR 2.76) in STEMI cohorts [18].

Our findings extend this evidence into a novel domain—functional cardiac recovery—as assessed by CMR-derived global longitudinal strain. Whereas prior investigations concentrated on event-free survival, we demonstrate for the first time that baseline potassium is independently associated with quantitative improvement in myocardial contractile function at 1 year post-STEMI.

It is important to note that while continuous variable analysis demonstrated a significant association (*p* = 0.041 in the prespecified primary model), the categorical tertile-based analysis showed a trend that approached but did not reach statistical significance (*p* for trend = 0.159). This suggests that the relationship, while consistent in direction, may be more subtle in magnitude than initially hypothesized, and warrants validation in larger cohorts.

The relationship we observed appeared approximately linear across the normal physiological range (3.4–4.5 mmol/L). This divergence from classic U-shaped mortality curves likely reflects fundamentally different biological mechanisms: mortality in the acute setting is driven primarily by arrhythmic events and electrophysiological toxicity (where extremes of potassium are hazardous), whereas long-term functional recovery involves cellular remodeling, inflammation, and myocardial energetics—processes that may benefit from higher-normal potassium levels without the threshold toxicity seen for electrical conduction.

Furthermore, our comprehensive electrolyte panel revealed that potassium uniquely predicts functional recovery, whereas sodium, chloride, magnesium, ionized calcium, and phosphorus showed no independent associations. This specificity is noteworthy given that magnesium has been investigated as a potential cardioprotective agent in AMI, though clinical trials (notably ISIS-4 with 58,050 patients) failed to demonstrate mortality benefit [19]. Importantly, our multivariate adjustment for baseline abs_GLS and 16 clinical covariates including discharge medications—a step rarely performed in traditional biomarker studies—was essential to reveal potassium’s independent prognostic value. Notably, potassium showed significant associations in both univariable analysis (r = 0.218, *p* = 0.017) and the primary multivariable analysis (β = 0.782, *p* = 0.041), with a consistent result in the mutually adjusted Model 2 robustness analysis (β = 0.866, *p* = 0.035).

### 4.3 Mechanistic Interpretation

The linear positive association between baseline potassium and long-term myocardial strain recovery, in contrast to the U-shaped relationship with acute mortality, suggests distinct mechanistic pathways beyond immediate electrophysiological toxicity.

Electrophysiological Stability and Repolarization Reserve: Potassium plays a fundamental role in maintaining cardiac electrophysiological integrity through ion channel subtypes, particularly the inward rectifier current (IK1) and ATP-sensitive potassium (KATP) channels [9,20]. Within the normal range, higher baseline potassium may enhance “repolarization reserve”—wherein IKr, IKs, and IK1 provide overlapping redundancy to ensure stable repolarization [21,22]. By increasing potassium channel conductance, it could reduce vulnerability to arrhythmogenic afterdepolarizations during the vulnerable post-infarction period, minimizing secondary ischemic injury from arrhythmia-related hemodynamic perturbations and preserving viable myocardium.

Mitochondrial Cardioprotection via KATP Channels: Mitochondrial ATP-sensitive potassium (mitoKATP) channels are pivotal mediators of ischemic preconditioning and cardioprotection against ischemia-reperfusion injury [23,24]. During ischemia, rising ADP/ATP ratios activate mitoKATP channels, triggering mild mitochondrial depolarization that reduces calcium overload, preserves redox balance, and inhibits mitochondrial permeability transition pore opening—collectively attenuating cardiomyocyte apoptosis and necrosis [23,24]. Higher baseline potassium may enhance mitoKATP activation capacity, thereby augmenting endogenous cardioprotective signaling during acute ischemic insult. This could explain our linear dose-response observation: whereas excessive potassium triggers electrophysiological toxicity (accounting for U-shaped mortality curves), moderate elevations within the normal range may selectively enhance mitoKATP-mediated cytoprotection without crossing the threshold for conduction abnormalities.

Modulation of Inflammatory Remodeling: Emerging evidence implicates potassium homeostasis in regulating innate immune responses relevant to post-infarction remodeling. Potassium efflux is a canonical trigger for NOD-like receptor family pyrin domain containing 3 (NLRP3) inflammasome activation, which drives interleukin-1β release, amplifying inflammatory cascades in infarcted myocardium [25,26]. Maintaining higher extracellular potassium concentrations may attenuate this pathological signaling by preserving intracellular stores and minimizing efflux-driven inflammasome assembly. In murine models, NLRP3 inhibition reduces infarct size, mitigates adverse ventricular remodeling, and preserves systolic function [26].

Acute STEMI induces a pronounced catecholamine surge that promotes intracellular potassium shift and transient hypokalemia [27], such that admission potassium may partly reflect the acute stress response rather than baseline potassium homeostasis. However, our findings are not readily explained by this mechanism alone. If lower potassium were simply a marker of greater ischemic stress, it should be associated with worse subsequent recovery; instead, higher admission potassium was associated with better 1-year GLS recovery. Furthermore, whereas prior large-scale studies have shown a U-shaped relationship between admission potassium and short-term outcomes after acute MI, including in-hospital mortality and ventricular arrhythmias [6], our endpoint was long-term myocardial functional recovery. Notably, the association remained independent after multivariable adjustment. Taken together, these data suggest that higher admission potassium may have a cardioprotective association beyond merely reflecting stress severity.

Importantly, these mechanistic hypotheses remain speculative in the absence of experimental validation. Our observational design cannot establish causality, and residual confounding by unmeasured factors (e.g., dietary patterns, neurohormonal activation states) may contribute to the observed association. Mechanistic elucidation will require translational investigations employing potassium supplementation trials with serial CMR endpoints and animal models with potassium clamping protocols to dissect causal pathways.

### 4.4 Clinical Implications

These findings carry potentially important clinical implications for risk stratification and hypothesis generation for future interventions in STEMI patients. First, baseline potassium concentration—a routinely measured, inexpensive biomarker—may serve as an early prognostic indicator for long-term functional recovery. Patients presenting with lower-normal potassium levels (e.g., 3.5–3.8 mmol/L) may exhibit attenuated strain improvement at 1 year compared to those with higher-normal levels, though the categorical analysis showed no significant trend (*p* = 0.159), suggesting that admission electrolyte profiles could complement existing risk models (e.g., GRACE, TIMI scores) for identifying individuals at elevated risk for incomplete myocardial recovery.

Second, our observations raise the hypothesis that optimizing potassium levels within the normal range—rather than merely correcting hypo- or hyperkalemia—might represent a modifiable target for improving post-infarction ventricular function. However, this remains strictly speculative pending randomized controlled trials. No contemporary randomized controlled trials (RCTs) have demonstrated that potassium supplementation or pharmacological potassium modulation causally improves CMR-derived strain recovery in STEMI patients. Importantly, aggressive potassium supplementation carries risks of iatrogenic hyperkalemia, particularly in patients receiving renin-angiotensin-aldosterone system inhibitors [28]. Therefore, while our findings are biologically intriguing, clinical translation requires prospective validation through trials designed to test whether targeted potassium optimization (e.g., maintaining 4.0–4.5 mmol/L) improves myocardial strain recovery without increasing adverse events.

Further consideration is warranted regarding the clinical significance of the observed effect size. In the prespecified primary model, each 1-SD increase in potassium (0.26 mmol/L) was associated with a 0.782-percentage-point higher 1-year abs_GLS. Although this difference may appear modest, an original cohort study in patients with heart failure with preserved ejection fraction reported that each 1-percentage-point worsening in signed GLS—equivalent to a 1-percentage-point decrease in abs_GLS—was associated with 10% higher odds of the composite of cardiovascular mortality or heart failure hospitalization [29]. Moreover, CMR-derived myocardial strain has demonstrated prognostic value following STEMI [10]. These findings support the potential prognostic relevance of relatively small differences in GLS; however, they do not establish that an intervention-induced improvement in GLS will reduce clinical events. Our study did not assess mortality or heart failure hospitalization, and prospective studies are required to determine whether potassium-associated differences in abs_GLS translate into improved clinical outcomes.

### 4.5 Strengths and Limitations

This study has several strengths. First, this appears to be the first investigation to systematically explore the association between within-normal-range potassium levels and CMR-derived myocardial strain recovery in STEMI patients. Second, our hypothesis-driven design—designating potassium as the primary exposure based on established mortality literature—minimizes the risk of HARKing and enhances interpretability. Third, statistical robustness was supported by consistent findings in the prespecified primary Model 1 (β = 0.782, *p* = 0.041) and the mutually adjusted Model 2 robustness analysis (β = 0.866, *p *= 0.035). Fourth, the use of CMR-derived global longitudinal strain provides superior sensitivity for detecting subclinical myocardial dysfunction compared to traditional LVEF.

However, several limitations warrant consideration. The study cohort was highly selected: only 120 of the 380 consecutive patients with STEMI treated with primary PCI at our hospital during the study period were included (31.6%). Selection and attrition bias cannot be excluded because 186 patients (48.9%) did not undergo the required baseline and/or 1-year CMR examinations, mortality data were unavailable, and more than 10% of potentially eligible patients were lost to follow-up; these factors may have affected the observed associations and limit their generalizability. First, our single-center retrospective design with predominantly male patients (94.2%) limits generalizability. Notably, the sex distribution in our cohort reflects a common challenge in STEMI-CMR research. In the large SWEDEHEART registry [30], men accounted for 41,664 of 62,048 patients with first-presentation STEMI (67.2%), whereas men comprised 94.2% of our CMR cohort. This difference underscores the limited generalizability of our predominantly male sample. Furthermore, the strict CMR follow-up protocol in our study (1-year interval) and requirements for breath-hold tolerance and renal function (for contrast administration) may disproportionately exclude older women with more comorbidities. Additionally, our single-center design may reflect regional and population-specific referral patterns. Consequently, our findings should be extrapolated to female STEMI patients with great caution; prospective multicenter validation studies specifically enrolling women are warranted to confirm that the potassium-myocardial recovery relationship observed in our predominantly male cohort extends to female patients. Second, our sample size (n = 120) constrained adequately powered subgroup analyses. Third, we assessed GLS as a surrogate endpoint rather than hard clinical outcomes such as mortality or heart failure hospitalization; while GLS deterioration has been linked to adverse long-term prognosis in multiple prior studies [10,11], whether interventions that improve strain translate to better clinical outcomes requires verification. Fourth, the observational design precludes causal inference—residual confounding by unmeasured factors (e.g., dietary potassium intake, genetic polymorphisms) may contribute to the observed association. Fifth, we measured potassium only at admission, which cannot capture subsequent fluctuations; a detailed discussion of the acute stress hypothesis is provided in Section 4.3. Nevertheless, our focus on baseline potassium reflects its immediate clinical utility for early risk stratification.

Sixth, while we used tertile categorization as a complementary analytic approach to enhance clinical interpretability, we acknowledge that any categorization of continuous variables involves some loss of statistical information. The categorical analysis showed a trend (*p* for trend = 0.159) that, although not reaching conventional statistical significance, was remarkably consistent with the significant findings from continuous variable analyses (*p* = 0.041). This consistency across different analytical approaches (continuous ANCOVA, restricted cubic splines, and tertile-based analysis) strengthens confidence in the robustness of the potassium-strain relationship and suggests that the findings are not artifacts of the analytical method chosen. The primary continuous variable analysis, which preserves all statistical information, remains the foundation of our conclusions.

Seventh, while feature-tracking CMR provides valuable and reproducible myocardial functional markers, several methodological limitations of GLS measurement in our study warrant acknowledgment. First, inter-vendor variability between different feature-tracking software packages has been shown to affect strain quantification results, and our study used a single software platform (Medis QStrain)—extrapolation to other platforms should be interpreted with caution. Second, GLS is sensitive to load conditions—it may be influenced by blood pressure or ventricular preload changes. Additionally, the relatively lower temporal resolution and frame rate settings of CMR cine imaging may also affect strain measurement precision. Finally, external mechanical factors such as thoracic morphology or chest wall geometry may influence myocardial motion tracking accuracy. Although these technical factors do not alter our primary conclusions, they should be considered when interpreting GLS results.

## 5. Conclusions

In this observational study, each 1-SD increase in baseline serum potassium (0.26 mmol/L) was associated with a 0.782-percentage-point increase in 1-year abs_GLS (95% CI: 0.042–1.521, *p* = 0.041) in the prespecified primary model adjusted for baseline abs_GLS and 16 clinical covariates. Higher-normal potassium levels were associated with better myocardial functional recovery, although the observational design precludes causal inference. These findings suggest that baseline serum potassium may serve as a readily available prognostic biomarker for risk stratification. Multicenter prospective studies and randomized controlled trials evaluating targeted potassium management strategies are needed to validate these findings and determine whether potassium optimization can improve post-infarction myocardial recovery.

## Data Availability

The datasets generated and analyzed during the current study are not publicly available due to institutional data protection policies but are available from the corresponding authors on reasonable request.

## Abbreviations

abs_GLS, absolute global longitudinal strain; AMI, acute myocardial infarction; ANCOVA, analysis of covariance; CI, confidence interval; CKD-EPI, Chronic Kidney Disease Epidemiology Collaboration; CMR, cardiac magnetic resonance; DBP, diastolic blood pressure; eGFR, estimated glomerular filtration rate; FDR, false discovery rate; GLS, global longitudinal strain; HR, hazard ratio; ICC, intraclass correlation coefficient; IQR, interquartile range; KATP, ATP-sensitive potassium channel; LAD, left anterior descending artery; LV, left ventricle/left ventricular; LVEF, left ventricular ejection fraction; MACE, major adverse cardiovascular events; MI, myocardial infarction; mitoKATP, mitochondrial ATP-sensitive potassium channel; NLRP3, NOD-like receptor family pyrin domain containing 3; PCI, percutaneous coronary intervention; RCS, restricted cubic splines; RCT, randomized controlled trial; SBP, systolic blood pressure; SD, standard deviation; STEMI, ST-segment elevation myocardial infarction; TIMI, Thrombolysis in Myocardial Infarction.
