# Supplementary material for: Baseline Serum Potassium Within the Normal Range Is Independently Associated With One-Year Myocardial Strain Recovery in Patients With ST-Segment Elevation Myocardial Infarction Treated With Primary Percutaneous Coronary Intervention: A Cardiac Magnetic Resonance Study
Source: Rev Cardiovasc Med. 2026 Aug 13;27(8):48335. doi: 10.31083/RCM48335 (PMC13540027; doi:10.31083/RCM48335)
Supplement: Supplementary file 1 [file 2153-8174-27-8-48335-s1.zip › Supplementary Material/Supplementary Material-V3.docx]

Supplementary Fig. 1. Study Flow Diagram Showing Patient Selection and Sequential Exclusions. Among 380 consecutive patients with STEMI who underwent primary PCI at the Affiliated Jiangyin Hospital of Nantong University between January 2020 and December 2023, 186 were excluded because baseline and/or 1-year CMR was unavailable, leaving 194 patients with both baseline and 1-year CMR. Of these, 58 were excluded because baseline electrolyte measurements were incomplete, leaving 136 patients. A further 16 patients were excluded because clinical covariate data were incomplete, resulting in a final analytic cohort of 120 patients. Boxes on the right represent excluded patients, whereas boxes in the main vertical pathway represent the remaining eligible cohort after each exclusion step. Patients lost to follow-up who consequently lacked 1-year CMR were included in the first exclusion category and were not counted again; therefore, no patient was counted more than once. Abbreviations: CMR, cardiac magnetic resonance; PCI, percutaneous coronary intervention; STEMI, ST-segment elevation myocardial infarction.

Supplementary Fig. 2. Baseline electrolyte distributions in the study cohort. This figure shows the distributions of six baseline electrolytes measured from venous blood samples collected at hospital admission before percutaneous coronary intervention (PCI): (A) sodium (mean ± SD, 139.51 ± 1.97 mmol/L), (B) potassium (3.92 ± 0.26 mmol/L), (C) chloride (103.05 ± 3.58 mmol/L), (D) magnesium (0.90 ± 0.04 mmol/L), (E) ionized calcium (1.02 ± 0.21 mmol/L), and (F) phosphorus (0.95 ± 0.11 mmol/L). Individual points represent individual patient measurements. In each panel, the box represents the interquartile range, the horizontal line within the box represents the median, the whiskers extend to the most extreme observations within 1.5 times the interquartile range, and the white diamond represents the mean. Laboratory reference-limit lines are not shown because institution- and analyzer-specific reference intervals could not be reliably verified from the available source documentation. Accordingly, the figure is presented solely as a descriptive summary of the observed baseline electrolyte distributions and is not intended to validate the measurements or imply that all observations were within normal reference limits. Abbreviations: PCI, percutaneous coronary intervention; SD, standard deviation.

Supplementary Fig. 3. Univariable associations between baseline electrolytes and Δabs_GLS. This figure presents six scatter plots examining univariable associations between baseline electrolyte concentrations and absolute global longitudinal strain change (Δabs_GLS = 1-year abs_GLS − baseline abs_GLS) in 120 STEMI patients: (A) sodium, (B) potassium, (C) chloride, (D) magnesium, (E) ionized calcium, and (F) phosphorus. Each panel displays individual patient data points, with the horizontal axis representing the baseline electrolyte concentration (mmol/L) and the vertical axis representing Δabs_GLS (percentage points). A purple regression line with 95% confidence interval (shaded area) illustrates the unadjusted linear relationship. Pearson correlation coefficients (r) and corresponding p-values are displayed in each panel. Panel B (potassium) demonstrates a significant positive correlation (r = 0.218, p = 0.017), indicating that higher baseline potassium is associated with greater myocardial strain improvement (more positive Δabs_GLS). All other electrolytes showed no significant univariable associations with Δabs_GLS (all p > 0.05): sodium (r = −0.048, p = 0.599), chloride (r = 0.036, p = 0.696), magnesium (r = 0.001, p = 0.991), ionized calcium (r = −0.006, p = 0.951), and phosphorus (r = 0.015, p = 0.875). These univariable findings were further evaluated in multivariable ANCOVA models adjusting for baseline abs_GLS and 16 clinical covariates (see Model 1 in table 2 of the main manuscript), where potassium remained the sole only electrolyte significantly associated of 1-year abs_GLS (β = 0.782, 95% CI: 0.042–1.521, p = 0.041). Abbreviations: abs_GLS, absolute global longitudinal strain; Δabs_GLS, absolute global longitudinal strain change; ANCOVA, analysis of covariance; CI, confidence interval; STEMI, ST-segment elevation myocardial infarction.

| Analysis | Potassium specification | N | β | 95% CI | p value |
| --- | --- | --- | --- | --- | --- |
| Primary continuous analysis | Per 1-SD increase (0.26 mmol/L) | 120 | 0.78 | (0.04,1.52) | 0.041 |
| Extreme-value sensitivity analysis | Per 1-SD increase after excluding K <3.0 or >5.0 mmol/L | 120 | 0.78 | (0.04,1.52) | 0.041 |
| Median dichotomization | High (>3.94 mmol/L) vs low (≤3.94 mmol/L) | 120 | 1.75 | (0.24, 3.25) | 0.023 |
| 4.0 mmol/L dichotomization | High (>4.0 mmol/L) vs low (≤4.0 mmol/L) | 120 | 1.44 | (−0.09, 2.96) | 0.065 |

**Supplementary Table 1. Sensitivity analyses of baseline serum potassium and 1-year abs_GLS.**

All models used 1-year abs_GLS as the outcome and adjusted for baseline abs_GLS and 16 clinical covariates: age, gender, diabetes, hypertension, smoking, Killip class, estimated glomerular filtration rate, systolic blood pressure, diastolic blood pressure, heart rate, symptom-to-balloon time, albumin, culprit artery, and discharge medications, including renin-angiotensin system inhibitors, β-blockers, and mineralocorticoid receptor antagonists. For the continuous analyses, potassium was standardized to 1 SD before model fitting, whereas 1-year abs_GLS was retained on its original percentage-point scale. Accordingly, the continuous β coefficients represent the percentage-point difference in 1-year abs_GLS per 1-SD increase in potassium (0.26 mmol/L). For the dichotomous analyses, β coefficients represent the adjusted difference in 1-year abs_GLS between the specified higher- and lower-potassium groups. No patients had potassium concentrations <3.0 or >5.0 mmol/L; therefore, the extreme-value sensitivity analysis included the same 120 patients and produced estimates identical to those of the full-sample continuous analysis. Results were directionally consistent across potassium specifications, although the 4.0 mmol/L dichotomization did not reach statistical significance. Abbreviations: abs_GLS, absolute global longitudinal strain; CI, confidence interval; SD, standard deviation.

**Supplementary Table 2. Univariable Pearson correlations between baseline electrolyte concentrations and Δabs_GLS.**

| Electrolyte | Pearson r | P value |
| --- | --- | --- |
| Sodium | -0.048 | 0.599 |
| Potassium | 0.218 | 0.017 |
| Chloride | 0.036 | 0.696 |
| Magnesium | 0.001 | 0.991 |
| Ionized calcium | -0.006 | 0.951 |
| Phosphorus | 0.015 | 0.875 |

Values are unadjusted Pearson correlation coefficients (r) and two-sided p values from 120 patients. Δabs_GLS was defined as 1-year abs_GLS minus baseline abs_GLS and was expressed in percentage points; a positive value indicates an increase in the absolute magnitude of GLS. Potassium was the only electrolyte significantly correlated with Δabs_GLS (r = 0.218, p = 0.017). Supplementary Fig. 3 presents the corresponding scatter plots with fitted linear regression lines and 95% confidence intervals. In the prespecified primary ANCOVA analysis reported in Table 2 of the main manuscript, which used 1-year abs_GLS as the outcome and adjusted for baseline abs_GLS and 16 clinical covariates, each 1-SD increase in potassium was associated with a 0.782-percentage-point higher 1-year abs_GLS (95% CI: 0.042 to 1.521, p = 0.041). Abbreviations: abs_GLS, absolute global longitudinal strain; ANCOVA, analysis of covariance; CI, confidence interval; Δabs_GLS, change in absolute global longitudinal strain from baseline to 1 year; SD, standard deviation.
